# Schwerstverletzungsartenverfahren – Einfluss der Ziffer 11 „Komplikationen“ sowie der COVID-19-Pandemie auf ein Haus der traumatologischen Maximalversorgung

**DOI:** 10.1007/s00113-023-01294-0

**Published:** 2023-02-06

**Authors:** Moritz F. Lodde, Moritz Freistühler, Julia Sußiek, Josef Stolberg-Stolberg, Steffen Roßlenbroich, J. Christoph Katthagen, Michael J. Raschke

**Affiliations:** Klinik für Unfall‑, Hand- und Wiederherstellungschirurgie, Universitätsklinikum Münster, Albert-Schweitzer-Campus 1, 48149 Münster, Deutschland

**Keywords:** Verletzungsartenverzeichnis, SAV – Schwerstverletzungsartenverfahren, Stationäre Heilverfahren, Berufsgenossenschaft, Deutsche Gesetzliche Unfallversicherung, Injury type catalogue, SAV—Severe injury type procedures, Injured patients in context of the German statutory accident insurance, Employers’ liability insurance association, German statutory accident insurance

## Abstract

**Einführung:**

Die Sicherstellung der besten Therapie – das Heilverfahren (HV) – ist Aufgabe der Deutschen Gesetzlichen Unfallversicherung (DGUV). Das Verletzungsartenverzeichnis ist das Mittel zur Lenkung des HV. Ziele der Arbeit sind die Auswertung der mittelfristigen Entwicklung der Fallzahlen im DAV, VAV und SAV, der Ziffer 11 „Komplikationen“ sowie des möglichen Einflusses durch die COVID-19-Pandemie.

**Methodik:**

Alle im SAV-Zentrum stationären DAV-, VAV- und SAV-Fälle von Januar 2019 bis Dezember 2021 wurden retrospektiv eingeschlossen. Die Fallzahlen vor und während der Lockdownmaßnahmen wurden verglichen. Der Case-Mix-Index, die Anzahl der durchschnittlichen Operationen, die Saalzeitminuten und die stationäre Verweildauer wurden analysiert.

**Ergebnisse:**

67 % aller 2007 stationär behandelten Versicherten gehören dem SAV an. 51 % aller SAV-Fälle sind der Ziffer 11 zuzuordnen. Im Bereich des Schultergürtels und Ellenbogens, an der Hand, am Kniegelenk und im Bereich des Sprunggelenks und Fußes sind viele Fälle der Ziffer 11 behandelt worden. Diese sind wirtschaftlich nur entsprechend der Fälle des VAV abgebildet. Durch die Lockdownmaßnahmen sind die Fallzahlen signifikant zurückgegangen. Das Verhältnis der Fallzahlen im DAV und VAV vs. SAV hat sich nicht verändert.

**Schlussfolgerung:**

Im Einzugsgebiet des vorliegenden SAV-Zentrums wird die Steuerung des HV in DAV, VAV und SAV erfolgreich verwendet. Der Großteil aller Fälle gehört dem SAV an. Hiervon wiederum ist mehr als die Hälfte der Ziffer 11 zuzuordnen. Der hohe Anteil an Komplikationsfällen der Ziffer 11 wirft die Frage nach der Notwendigkeit einer strukturellen Anpassung des HV auf. Die Kommentierung des aktualisierten Verletzungsartenverzeichnisses bietet entscheidende klärende Definitionen, jedoch sollte die Übersichtlichkeit erhalten bleiben.

**Graphic abstract:**

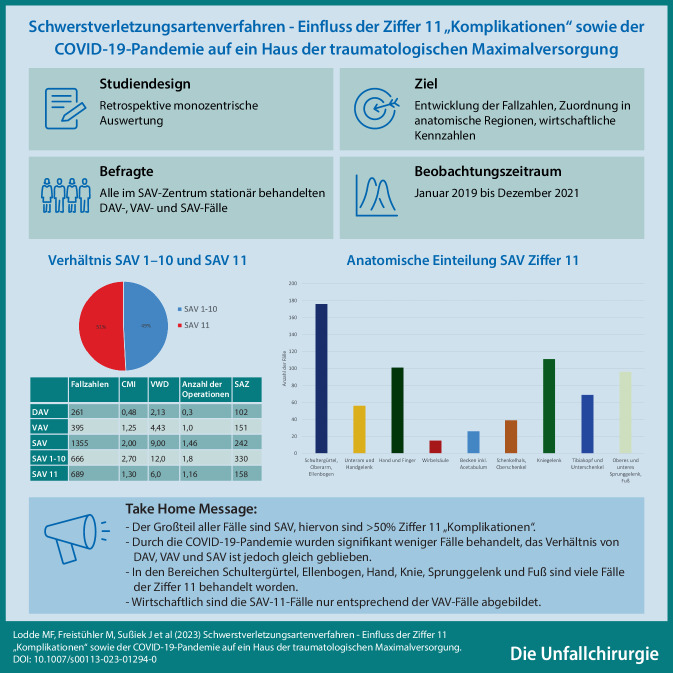

## Abstract (en)

**Introduction:**

Ensuring the best treatment options for injured patients (healing process, *Heilverfahren*, HV) is the main goal and responsibility of the German statutory accident insurance (DGUV). The injury type catalogue is the tool to guide the HV. The development of the number of cases treated in a center for severe injury type procedures, the effects of the cipher 11 “complications” of the catalogue and the effects of the COVID-19 pandemic are presented.

**Methods:**

A retrospective study of all patients treated in the context of the DGUV from January 2019 to December 2021 was carried out. The number of cases before and during the legal lockdown actions were compared. The case mix index, the mean number of operations, the mean time in the operating room and the hospital stay were analyzed. Additionally, the cases under the cipher 11 were classified according to the specific anatomical regions.

**Results:**

Out of 2007 cases 67% were classified as severe injury type procedures (SAV). Of these cases 51% were categorized to the cipher 11 of the injury type catalogue. Complications were observed particularly in the anatomical regions of the shoulder girdle, elbow, hand, knee, ankle joint and foot. These complex cases are economically not sufficiently represented. During the governmental COVID-19 lockdown actions, the number of patients treated in the context of the DGUV significantly decreased.

**Conclusion:**

The injury type catalogue is used effectively in the catchment area of the present trauma center. Most of the cases treated in the present trauma center are severe injury type procedures; however, more than half of these cases are classified as complications. This development might show the need for a structural change or an adjustment of the HV. The current comments on the injury type catalogue offer important definitions and specifications; however, the conciseness of the entire catalogue should be maintained.

Ein Aufgabenbereich der Deutschen Gesetzlichen Unfallversicherung (DGUV) besteht in der Sicherstellung einer optimalen medizinischen Behandlung von verunfallten Personen und von Komplikationen. Durch eine ressourcenorientierte Steuerung des Heilverfahrens (HV) soll der Gesundheitszustand dieser Personen vollständig bzw. bestmöglich wiederhergestellt werden [3]. Das spezifizierte und aktualisierte Verletzungsartenverzeichnis kann als Instrument zur Lenkung des HV verstanden werden [3].

## Das Heilverfahren

Von der DGUV wurden in Zusammenarbeit mit anderen Akteuren aus dem medizinischen Sektor das ambulante und stationäre Durchgangsarztverfahren (DAV), Verletzungsartenverfahren (VAV) und Schwerstverletzungsartenverfahren (SAV) entwickelt. Seitdem werden je nach Art und Komplexität der Gesundheitsschäden der betroffenen Personen sowie Komplikationen im HV medizinische Akteure in das HV eingebunden, welche eine entsprechende Beteiligung der Verfahren vorweisen können [1, 7]. Dabei müssen die am Schwerstverletzungsartenverfahren (SAV) beteiligten Kliniken den höchsten Behandlungsstandard gewährleisten.

### Die Kommentierung des aktuellen Verletzungsartenverzeichnisses

In der am 27.07.2021 veröffentlichten Kommentierung zum aktualisierten Verletzungsartenverzeichnis heißt es, dass die Versicherten in die am nächsten geeignete und am HV beteiligte Klinik der entsprechenden Versorgungsstufe verlegt werden müssen [3]. Das Verletzungsartenverzeichnis mit den Ziffern 1 bis 10 bezieht den Zeitraum zwischen Unfalltag bis 4 Monate nach Unfall ein [3]. Eine besondere Stellung nimmt die Ziffer 11 „Komplikationen“ ein [5].

Hierunter fallen:

- Infektion (11.1),
- Defektheilung des Weichteilmantels (11.2),
- Notwendigkeit von Revisionseingriffen (11.3),
- Verletzungsfolgezustände beim Kind (11.4),
- spezielle Komplikationen wie komplexes regionales Schmerzsyndrom (11.5).

Unabhängig vom Zeitpunkt gilt für Komplikationen, dass sie der Ziffer 11 zugeordnet werden. Im HV entstandene Komplikationen dürfen nur in SAV-Krankenhäusern behandelt werden. Posttraumatische Arthrosen, die eine endoprothetische Versorgung auch Jahre nach dem initialen Unfall erfordern, fallen unter die Ziffer 11.3 und sind dem SAV zuzuordnen [3]. Die singuläre Verletzung eines der Nerven N1–N4 und N10 an der Hand führt zur Zuordnung zum SAV. Eine tiefgehende Infektion (SAV 11.1) liegt vor, wenn Gewebeschichten unterhalb der Subkutis befallen sind [3]. Unter 11.2 „Defektheilung des Weichteilmantels“ wird die entstellende Ästhetik definiert [3]. Ausgedehnte und aufwendige Revisionseingriffe (SAV 11.3) sind periprothetische Frakturen, Notwendigkeit von Lappen- oder Nervenplastik, die Interposition von Bandplastiken, die Behandlung von Infektsituationen und Achskorrekturen. Ein komplexes regionales Schmerzsyndrom der Hand oder Phantomschmerzen fällt/fallen unter Punkt 11.5.

## Ziele der Arbeit

Die Hypothese, dass die Einführung der Ziffer 11 mittelfristig zu einer Zunahme der SAV-Fälle geführt hat, wird geprüft. Es erfolgt die Einteilung der Ziffer-11-Fälle „Komplikationen“ in anatomische Regionen und die wirtschaftliche Analyse. Neben der Überarbeitung des SAV-Katalogs mit nun vorliegender umfassender Kommentierung hat auch die COVID-19-Pandemie (SARS-CoV-2) mögliche bis dato weitgehend unklare Auswirkungen auf das HV. Ebenso erfolgen die kritische Beurteilung des Schwerstverletzungsartenverzeichnisses und die Darstellung möglicher Verbesserungen.

## Methodik

Alle im SAV-Zentrum stationär aufgenommenen DAV-, VAV- und SAV-Fälle aus dem Zeitraum Januar 2019 bis Dezember 2021 wurden retrospektiv entsprechend dem kommentierten aktualisierten Verletzungsartenverzeichnis ausgewertet [3, 5]. Neben der Darstellung der Fallzahlen wurde ein möglicher Einfluss der COVID-19-Pandemie untersucht. Hierzu wurden die Fallzahlen von Januar 2019 bis März 2020 mit den Fallzahlen von April 2020 bis Dezember 2021 verglichen. Dies erfolgte aufgrund der bundesweit durchgeführten Lockdownmaßnahmen und Einschränkungen ab dem 2. Quartal 2020 mit Erklärung zur Pandemie. Der Case-Mix-Index (CMI), die Anzahl der durchschnittlichen Operationen, die Saalzeitminuten und die stationäre Verweildauer für alle DAV, VAV und SAV-Fälle wurden analysiert. Zur besseren Vergleichbarkeit wurden alle Fälle in das DRG-System 2021 gruppiert. Anschließend erfolgten die Subgruppenanalyse für die SAV-Fälle 1–10 vs. SAV-Fälle 11 und die Zuordnung der SAV-Fälle 11 in anatomische Regionen. Die statistische Auswertung erfolgte mit IBM SPSS Statistics software (Version 26.0, International Business Machines, Armonk, NY, USA). Nach Testung auf Normalverteilung erfolgte der Kruskal-Wallis-Test mit der Dunn-Bonferroni-Korrektur oder der Mann-Whitney‑U Test mit *p* < 0,05.

## Ergebnisse

**Figure 1:**
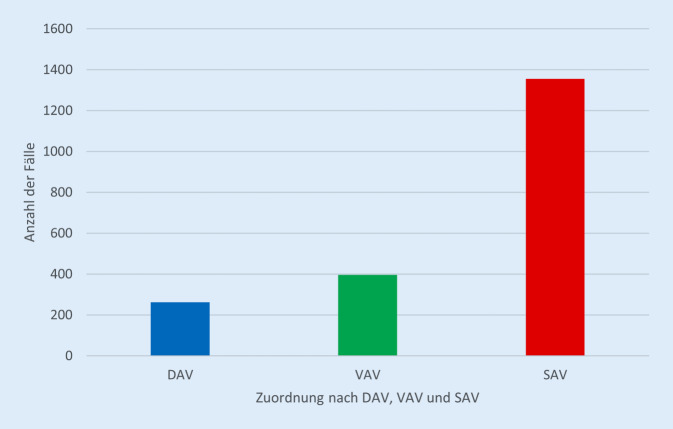

Im Untersuchungszeitraum wurden 2007 der im stationären HV behandelten Patienten im Haus der traumatologischen Maximalversorgung eingeschlossen. Von diesen 2007 Patienten gehören 13 % (261 Fälle) dem DAV, 20 % (395 Fälle) dem VAV und 67 % (1351 Fälle) dem SAV an (Abb. 1).

**Figure 2:**
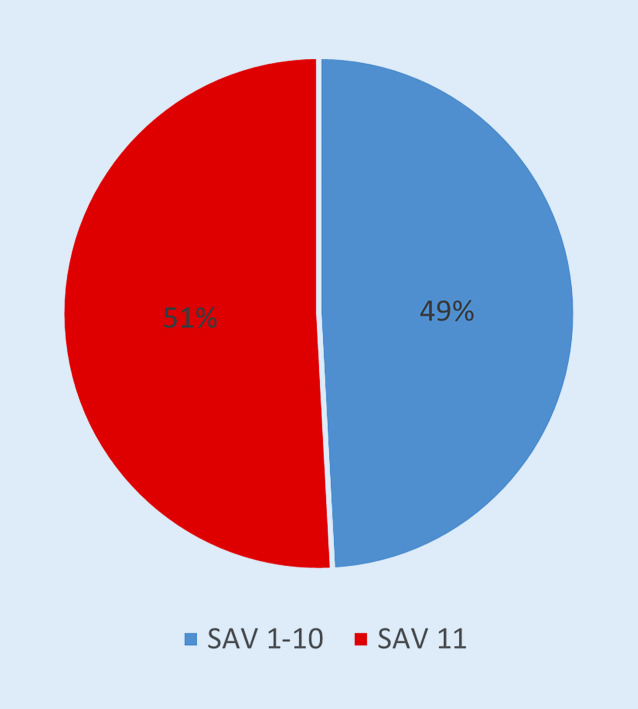

Von den 1351 SAV-Fällen sind 51 % (685 Fälle) SAV Ziffer 11 zuzuordnen (Abb. 2).

**Figure 3:**
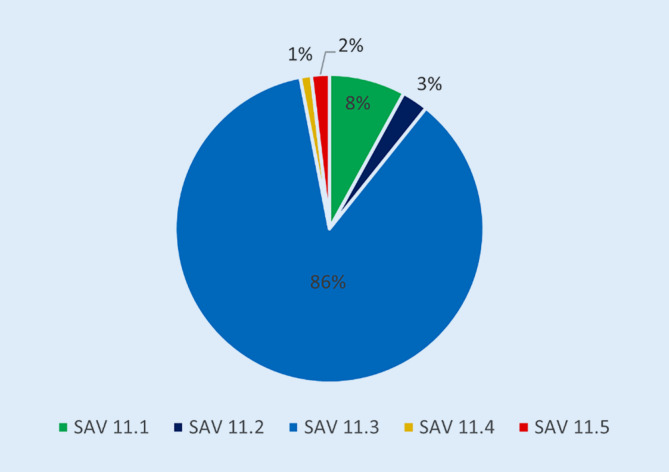

Von den 685 Fällen der Ziffer 11 fallen 8 % (55 Fälle) auf die Ziffer 11.1 „Infektionen/infektiöse Komplikationen“, 3 % (19 Fälle) auf die Ziffer 11.2 „Defektheilung des Weichteilmantels“, 86 % (590 Fälle) auf die Ziffer 11.3 „Notwendigkeit von Revisionseingriffen“, 1 % (8 Fälle) auf die Ziffer 11.4 „Verletzungsfolgezustände beim Kind“ und 2 % (13 Fälle) auf die Ziffer 11.5 „spezielle Komplikationen und Unfallfolgen“ (Abb. 3).

**Figure 4:**
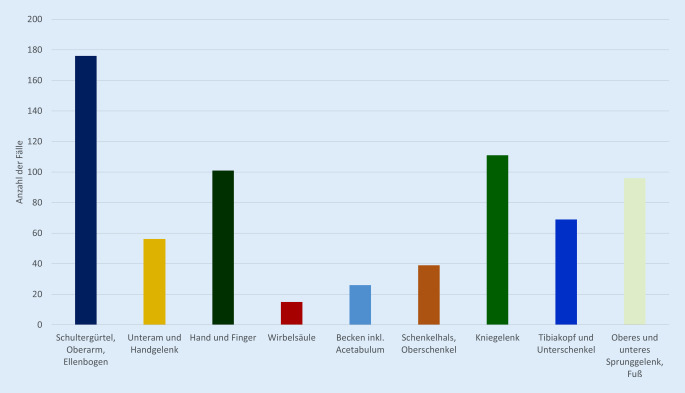

Die Auswertung nach anatomischer Region zeigt einen hohen Anteil an Komplikationen im Bereich des Schultergürtels und Ellenbogens, an der Hand, am Kniegelenk und im Bereich des Sprunggelenks und Fußes (Abb. 4).

Der mittlere CMI für alle SAV-Fälle im Untersuchungszeitraum beträgt 2,00. Der CMI der VAV-Fälle ist 1,25 und der CMI der DAV-Fälle 0,48 (Tab. 1). Signifikant unterscheiden sich der CMI zwischen dem SAV und DAV (*p* = 0,022). Die Subgruppenanalyse zeigt einen CMI für die SAV-Fälle 1–10 von 2,69 und für die SAV-Fälle 11 von 1,30 (*p* = 0,05).

**Table 1:** 

	Fallzahlen	CMI	VWD	Anzahl der Operationen	SAZ
*DAV*	261	0,48	2,13	0,3	102
*VAV*	395	1,25	4,43	1,0	151
*SAV*	1351	2,00	9,00	1,46	242
SAV 1–10	666	2,70	12,0	1,8	330
SAV 11	689	1,30	6,0	1,16	158

*DAV* Durchgangsarztverfahren, *VAV* Verletzungsartenverfahren, *SAV* Schwerstverletzungsartenverfahren

Die mittlere stationäre Verweildauer für alle SAV-Fälle im Untersuchungszeitraum ist 9,0 Tage. Die Verweildauer für alle DAV-Fälle ist 2,1 Tage und für alle VAV-Fälle 4,4 Tage. Signifikant unterscheiden sich die Verweildauern zwischen dem SAV und DAV (*p* = 0,020). Die SAV-Fälle 1–10 haben eine Verweildauer von 12,1 Tagen und die SAV-Fälle 11 von 6,0 Tagen (*p* = 0,05).

Die Anzahl an Operationen während des stationären Aufenthalts für das DAV ist 0,4. Für das VAV beträgt die Anzahl der Operationen 1,0 und für das SAV 1,5. Der Unterschied zwischen dem DAV und SAV ist signifikant (*p* = 0,018). Die SAV-Fälle 1–10 haben eine Anzahl von 1,8 Operationen und die SAV-Fälle der Ziffer 11 von 1,2 (*p* = 0,043).

Die mittleren Saalzeitminuten für das DAV sind 103 min, für das VAV 151 min und für das SAV 240 min (Tab. 1). Signifikant unterscheiden sich die Saalzeiten zwischen dem SAV und DAV (*p* = 0,022). Die SAV 1–10 Fälle haben eine Saalzeit von 329 min und die SAV-11 Fälle eine Saalzeit von 157 min (*p* = 0,046).

Die Auswertung der Fallzahlen pro Quartal im HV vor Beginn der COVID-19-Pandemie und während der COVID-19-Pandemie zeigt, dass es zu einem signifikanten Rückgang aller stationären Fälle des HV gekommen ist (Median 192 Fälle vs. 147 Fälle/Quartal, *p* = 0,003).* In den ersten beiden Quartalen 2019 wurden 196 Patienten, im dritten Quartal 2019 183 und im vierten Quartal 2019 192 Patienten stationär behandelt. Im ersten Quartal 2020 vor Beginn der Pandemieerklärung wurden 181 Patienten stationär behandelt. Im zweiten Quartal 2020 bestand mit 144 behandelten Patienten ein deutlicher Rückgang der aller stationären Fälle des HV. In dem dritten Quartal (170 Fälle) und vierten Quartal 2020 (131 Fälle) sowie im ersten Quartal (180 Fälle), zweiten Quartal und dritten Quartal (147 Fälle) und vierten Quartal 2021 (144 Fälle) konnte dieser signifikante Rückgang beobachtet werden*. Vor Beginn der Lockdownmaßnahmen war der Median der SAV-Fälle pro Quartal mit 128 signifikant höher als während der Lockdownmaßnahmen (102 Fällen, *p* = 0,005). Der Anteil der SAV-Fälle der Ziffer 11 hat sich von 0,57 auf 0,46 nicht signifikant reduziert (*p* = 0,43). Das Verhältnis zwischen dem DAV und VAV gegenüber dem SAV hat sich während der Lockdownmaßnahmen nicht geändert (Median 64 Fälle DAV/VAV und 128 Fälle SAV, Verhältnis 0,5, vs. Median 48 Fälle DAV/VAV und 102 Fälle SAV, Verhältnis 0,47, *p* > 0,05).

## Diskussion

### Die Fallzahlen in DAV, VAV, SAV

Im Untersuchungszeitraum sind die Fallzahlen des SAV mit einem Anteil von 67 % deutlich höher als die Fallzahlen des DAV und VAV und stimmen mit der Auswertung der kurzfristigen Fallzahlentwicklung nach Einführung des neuen Verletzungsartenverzeichnis überein [5]. Die mehrstufige Steuerung des HV wird im Einzugsgebiet des hier untersuchten traumatologischen Maximalversorgers erfolgreich angewendet. Dies ist an dem hohen Anteil der Fallzahlen des SAV abzuleiten.

### Die Fallzahlen im SAV und die Ziffer 11 „Komplikationen“

Von allen SAV-Fällen sind 51 % der Ziffer 11 zugeordnet. Dies spiegelt den Anspruch der gesetzlichen Unfallversicherung wider, „frühzeitig mit allen geeigneten Mitteln die körperliche und geistige Gesundheit der Unfallverletzten wiederherzustellen“ [3]. Auffällig ist der große Anteil der Fälle, die der Ziffer 11.3 „Notwendigkeit von Revisionseingriffen“ zuzuordnen sind (Abb. 3). Die Analyse nach anatomischer Region zeigt einen hohen Anteil an Komplikationen im Bereich des Schultergürtels und Ellenbogens, an der Hand, am Kniegelenk und im Bereich des Sprunggelenks und Fußes. Der niedrige Anteil an Komplikationen im Bereich der Wirbelsäule und des Beckens kann möglicherweise dadurch erklärt werden, dass diese Verletzungen bereits initial in Krankenhäusern der Maximalversorgung bzw. spezialisierten Einrichtungen versorgt werden. Eine entscheidende Frage ist, wie der hohe Anteil der Fälle der Ziffer 11 „Komplikationen“ reduziert werden kann. Ein möglicher Lösungsansatz ist die Vergabe einzelner VAV- und SAV-Zulassungen an am HV beteiligten Krankenhäuser entsprechend der höchsten Qualitätskriterien einzelner Fachgesellschaften im Rahmen von Zertifizierungsprozessen. Die Auswertung, ob der hohe Anteil der Fälle 11 „Komplikationen“ schicksalhaft ist, oder ob ggf. strukturelle Änderungen notwendig sind, steht noch aus.

## Wirtschaftliche Analyse

Die wirtschaftliche Analyse zeigt für die Fälle des SAV den höchsten CMI, die längste mittlere Verweildauer, die höchste mittlere Anzahl an Operationen und die meisten mittleren Saalzeitminuten (Tab. 1). Auffallend ist der Vergleich zwischen den Fällen des VAV und den Fällen der Ziffer 11 im SAV. Bei nur leicht höherem CMI des SAV11-Fälle (+4,8 %) ist die Verweildauer für die Fälle der Ziffer 11mit 1,6 Tagen länger (+36,4 *%). Die Vorhaltekosten, die für die Versorgung der SAV-Fälle notwendig sind, scheinen im DRG-System, einem System der reinen Betriebskostenfinanzierung, nur bedingt refinanziert*. Ein Zuschlagssystem zur weiteren Finanzierung ist bisher nicht flächendeckend bzw. nach Kenntnis der Autoren nur für Kliniken in Trägerschaft der Berufsgenossenschaften eingeführt worden. Die an der InEK-Kalkulation teilnehmenden Kliniken in berufsgenossenschaftlicher Trägerschaft lieferten in der Vergangenheit nur Kostendaten für Fälle, bei denen die DGUV nicht Kostenträger ist [2]. *Für ein Zuschlagssystem können auch wiederkehrende Analysen angesehen werden, welche die Versorgung von Schwerstverletzten als eher wirtschaftlich unattraktiv identifizieren *[6, 8].

## Auswirkung der COVID-19-Pandemie

**Figure 5:**
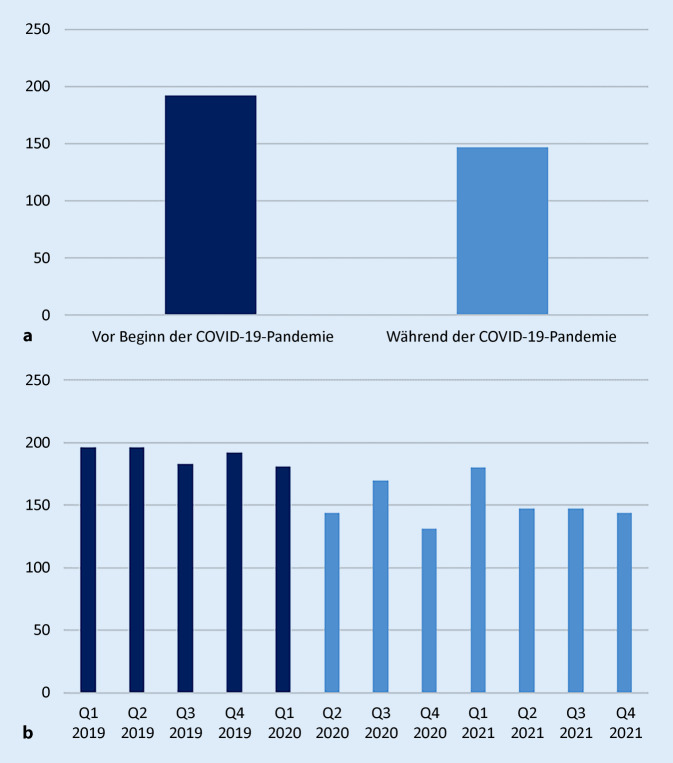

Die Fallzahlen vor dem ersten bundesweiten Lockdown aufgrund der COVID-19-Pandemie waren signifikant höher als nach dem Beginn der erstmaligen Lockdownmaßnahmen (Abb. 5). Dieses Ergebnis ist mit den Resultaten anderer Studien vergleichbar [4]. Interessanterweise ist das Verhältnis von Fällen in DAV und VAV gegenüber den Fällen im SAV gleichgeblieben. Auch der Anteil der SAV-Fälle der Ziffer 11 am SAV hat sich nicht geändert. Die vorübergehende Schließung von industriellen Produktionsstätten und Reduzierung des analogen Schulunterrichts – beides mit einem Rückgang der Wegeunfälle – könnten Gründe für den signifikanten Rückgang der Fallanzahl sein. Auch die Zunahme der Homeoffice-Tätigkeit ist eine mögliche Erklärung.

## Die Kommentierung des Verletzungsartenverzeichnisses aus Sicht des Traumazentrums der Maximalversorgung

Die Kommentierungen des Verletzungsartenverzeichnisses sind zur Einteilung der Verletzungen hilfreich. Die Zuordnung der Fälle für die Ziffer 11 ist weiterhin nicht eindeutig. So fallen unter 11.1 Infektionen und unter 11.3 die operative Therapie von Infektsituationen [3]. Deswegen wurden in der vorliegenden Arbeit operative therapierte infektiöse Komplikationen zu Punkt 11.3 eingeteilt. Gleiches gilt für kindliche Komplikationen, die sowohl unter 11.3 als auch unter 11.4 eingeordnet werden können. Dies erklärt die niedrige Anzahl an Fällen 11.4 in der vorliegenden Arbeit. Bei Vorliegen von polytraumatisierten Patienten sollte außerdem geklärt werden, welche Ziffer des SAV zur Einteilung der Behandlung zu nutzen ist. Bei einem ISS ≥ 25 und gleichzeitigem Vorliegen einer instabilen Beckenringfraktur können die Ziffer SAV 9.3 oder SAV 10.1 verwendet werden.

Aus Sicht des Traumazentrums der Maximalversorgung sind Arbeitsunfälle mit schweren Abdominalverletzungen bisher nicht ausreichend abgebildet. Die Entwicklung einer Ziffer im SAV für schwerste abdominelle Verletzungen des Erwachsenen ist notwendig, um auch für diese Patienten die bestmögliche Therapie zu ermöglichen.

Auch wenn die Kommentierung des Verletzungsartenverzeichnisses eine verbesserte Einteilung der Fälle ermöglicht, muss die weitere Zunahme des Umfangs kritisch gesehen werden. Im klinischen Alltag muss für alle am HV beteiligten Kliniken die schnelle, eindeutige und reproduzierbare Einteilung der Verletzungen und der Komplikationen möglich sein. Dann kann das Verletzungsartenverzeichnis erfolgreich eingesetzt werden.

## Zusammenfassung

Seit Einführung des aktualisierten Verletzungsartenverzeichnisses funktioniert die mehrstufige Steuerung des HV im Einzugsgebiet des vorliegenden traumatologischen Maximalversorgers erfolgreich. Der höchste Anteil aller Fälle gehört dem SAV an. Die Anzahl der Fälle 11 „Komplikationen“ und insbesondere die Fälle der Ziffer 11.3 bilden einen Großteil aller Fälle im SAV. Im Bereich des Schultergürtels und Ellenbogens, an der Hand, am Kniegelenk und im Bereich des Sprunggelenks und Fußes sind viele Fälle der Ziffer 11.3 zuzuordnen. Die Erlöse dieser Fälle sind bei längerer stationärer Verweildauer und leicht höheren Saalzeiten mit Fällen des VAV zu vergleichen. Die Behandlung der Fälle der Ziffer 11 im SAV erscheint wirtschaftlich nicht ausreichend abgebildet. Seit den bundesweiten Lockdownmaßnahmen im Rahmen der COVID-19-Pandemie sind die Fallzahlen im HV bei gleichem Anteil an Fällen im DAV, VAV und SAV signifikant geringer. Die Kommentierung des aktuellen Verletzungsartenverzeichnisses aus Juli 2021 liefert wichtige Definitionen und verbessert die Steuerung des HV weiter.

## Fazit für die Praxis

- Im Einzugsgebiet des vorliegenden Maximalversorgers wird die mehrstufige Steuerung des Heilverfahrens (HV) erfolgreich verwendet.
- 67 % aller 2007 stationär behandelten Versicherten gehören dem SAV an.
- 51 % aller SAV-Fälle sind der Ziffer 11 „Komplikationen“ zuzuordnen.
- Im Bereich des Schultergürtels und Ellenbogens, an der Hand, am Kniegelenk und im Bereich des Sprunggelenks und Fußes sind viele Fälle der Ziffer 11.3 behandelt worden.
- Die Fälle der Ziffer 11 erscheinen wirtschaftlich unattraktiver.
- Durch die Lockdownmaßnahmen im Rahmen der COVID-19-Pandemie sind die Fallzahlen im Heilverfahren signifikant zurückgegangen; das Verhältnis von DAV, VAV, SAV ist gleich geblieben.
- Aus Sicht des traumatologischen Maximalversorgers sollte eine Ziffer im SAV für schwerste abdominelle Verletzungen eingeführt werden.
- Die Kommentierung des aktualisierten Verletzungsartenverzeichnis bietet entscheidende klärende Definitionen; die Prägnanz sollte erhalten bleiben.
